# Evaluation of *FGF10* as a candidate gene for high myopia in a Han Chinese population

**DOI:** 10.1186/s40662-019-0158-x

**Published:** 2019-10-30

**Authors:** Lingxi Jiang, Dongyan Luo, Tingting Wang, Rui Zheng, Yaru Zhai, Xiaoqi Liu, Bo Gong, Zhengzheng Wu, Yin Yang, Zhenglin Yang, Yi Shi

**Affiliations:** 1 Sichuan Provincial Key Laboratory for Human Disease Gene Study, Department of Clinical Laboratory, Sichuan Academy of Medical Sciences & Sichuan Provincial People’s Hospital, School of Medicine, University of Electronic Science and Technology of China, 32 the First Ring Road West 2, Chengdu, 610072 Sichuan China; 2 Department of Ophthalmology, Sichuan Academy of Medical Sciences & Sichuan Provincial People’s Hospital, Chengdu, China; 3 Chinese Academy of Sciences Sichuan Translational Medicine Research Hospital, Chengdu, Sichuan China

**Keywords:** High myopia, Extreme myopia, Association study, Fibroblast growth factor 10

## Abstract

**Background:**

*Fibroblast growth factor 10* (*FGF10*) is implicated in the growth and development of the eye. Four singles nucleotide polymorphisms (SNPs) in the *FGF10* gene (including rs1384449, rs339501, rs12517396 and rs10462070) were found to be associated with extreme myopia (EM, refractive error ≤ − 10.0 diopters) in Japanese and Chinese Taiwan population. This case-control association study was conducted to explore the relationship between these four SNPs and high myopia in a western Chinese population.

**Methods:**

A total of 869 high myopia patients (HM, including 485 EM patients) and 899 healthy controls were recruited. These four SNPs were genotyped using the ABI SNaPshot method. Five genetic models (allelic, homozygous, heterozygous, dominant, and recessive) were applied to further evaluate the possible correlation between the SNPs and high myopia. The linkage-disequilibrium block (LD) structure was tested by Haploview Software.

**Results:**

In our study, no statistically significant differences were found between HM/EM patients and controls after Bonferroni multiple-correction (*P* > 0.05) in the allele frequencies of these four SNPs in the *FGF10* gene. We further found that rs12517396AA and rs10462070GG carriers showed a decreased risk of HM/EM compared with rs12517396AC + CC and rs10462070GA + AA carriers (*P* = 0.045, OR = 0.366; *P* = 0.021, OR = 0.131; *P* = 0.03, OR = 0.341; *P* = 0.015, OR = 0.122; respectively). Additionally, rs12517396AA and rs10462070GG carriers showed the same decreased risk of HM/EM compared with rs12517396CC and rs10462070AA carriers (*P* = 0.048, OR = 0.370; *P* = 0.023, OR = 0.133; *P* = 0.032, OR = 0.346; *P* = 0.017, OR = 0.126). However, these significant associations between rs12517396/rs10462070 and HM/EM disappeared after Bonferroni multiple-correction (*P* > 0.05).

**Conclusion:**

Our findings indicate that rs12517396 and rs10462070 had marginal association with HM and EM. The other two common polymorphisms in *FGF10* unlikely have significant effects in the genetic predisposition to HM/EM in western Chinese population. Further replication studies are needed to validate our findings in both animal models and human genetic epidemiologic studies.

## Background

Amongst the myriad of human eye diseases, myopia has one of the highest incidences. It is a serious health problem in the world and has heavy economic and financial burden to the society [1]. High myopia (HM), especially Extreme myopia (EM) may disrupt vision and induce pathological ocular changes. HM is defined as spherical equivalent (SE) ≤ − 6.00 diopters (D) and axial length (AL) ≥ 26 mm. EM is characterized by an SE ≤ − 10.00 D and extreme AL ≥ 30 mm [2]. Both HM and EM are significant risk factors for pathological ocular diseases and can cause many myopia complications such as choroidal neovascularization, glaucoma, retinal detachment, myopic macular degeneration and so on [3]. Many previous studies confirmed that the incidence of myopia increased remarkably in the last 30 years, especially in Southeast Asia [4, 5]. In Taiwan, more than 80% of young adults suffer from myopia [6–8]. However, the etiology and mechanism of myopia development are still unclear [9]. Family-based linkage analyses tested dozens of myopia regions [10, 11] and genome-wide association studies (GWAS) confirmed the complex inheritance of refractive error and identified over 150 gene loci with myopia. Consequently, some candidate genes of myopia have been reported, such as *ZNF644* [12–16], *CCDC111* [17], *LRPAP1* [18], *P4HA2* [18], *SLC39A5* [19].

Fibroblast growth factor 10 (*FGF10*) belongs to FGFs family and participates in the growth and development of different cells and organs, affecting the proliferation of ocular cells and other tissues [20]. In FGFs family, *FGF2* and *FGF10* are suggested to regulate enzyme activity during fat metabolism [21]. Notably, *FGF10* is abundantly expressed in the retina and sclera of mouse and human beings and plays an important role in ocular tissues. For example, the form-deprivation myopia (FDM) mouse model was used to demonstrate an increasing mRNA expression of FGF10 in FDM-treated eyes [22], suggesting that *FGF10* can be considered a candidate gene for myopia. Furthermore, four single nucleotide polymorphisms (SNPs) in *FGF10*, rs1384449, rs339501, rs12517396 and rs10462070, were reported to be associated with EM in the East Asians (Japanese and population in Taiwan, China) [23, 24]. In this study, we investigated whether these SNPs were significantly associated with HM/EM in a western Han Chinese population involving 869 unrelated high myopia patients and 899 unrelated healthy controls.

## Methods

### Study subjects

Healthy individuals were recruited at the health management center of Sichuan Provincial People’s Hospital. Their spherical equivalent were from − 1.0 to + 1.0 diopter sphere (DS) and had no evidence of disease in both eyes. All healthy controls were unrelated to individuals with high myopia.

Patients with high myopia were recruited at the clinic and ward of the Ophthalmic department of Sichuan Provincial People’s Hospital. All myopia subjects underwent standard visual acuity (including uncorrected and best-corrected) and B-ultrasonography to measure the diagnosis value and axial length. The diagnosis for high myopia (or extreme myopia) in this study the spherical equivalent should be ≤ − 6.0 (or − 10.0) DS in at least one eye and the axial length of the eye globe should be ≥26.0 (or 30.0) mm. High myopia can cause many myopia complications such as some fundus pathological changes. Therefore, all subjects underwent other ophthalmologic examinations too, including slit-lamp biomicroscopic examination, optical coherence tomography, dilated pupillary indirect ophthalmoscopic examination, and intraocular pressure examination. Individuals who had undergone ocular procedures or had other symptoms besides high myopia were excluded from this study. A total of 869 unrelated patients with HM (including 485 EM) and 899 normal controls were enrolled in this study (Table 1).

**Table 1:** Characteristics of controls and high myopia (HM) and extreme myopia (EM) patients in the study

Group	Number	Average age (years)^*^	Gender		Refractive errors (diopter)^*^		Axial length (mm)^*^	
			Male	Female	OD	OS	OD	OS
Control	899	55.92 ± 19.13	484	415				
High myopia	869	41.60 ± 20.57	481	388	−13.04 ± 6.43	−12.85 ± 6.39	29.68 ± 3.46	29.51 ± 2.42
Extreme myopia	485	42.13 ± 20.64	216	269	−14.92 ± 6.70	−14.63 ± 6.65	29.96 ± 2.95	29.77 ± 2.27

^*^ ±: standard deviation; *OD* = right eye, *OS* = left eye

### SNP selection and genotyping

In this study, we investigated 4 SNPs of the *FGF10* gene, including rs339501 and rs1384449 which are associated with EM in the Chinese population in Taiwan and 3 SNPs (rs339501, rs10462070 and rs12517396) which are related to EM in a Japanese population. Venous blood from 1768 subjects were collected in an EDTA tube. Total genomic DNA was obtained through serial phenol-chloroform extraction and ethanol precipitation. Four specific SNPs sites were amplified by ABI 2720 Thermal cycler machine and dye terminator-based SNaPshot method (Applied Biosystems, Foster City, CA) were used to genotype SNPs. All the products were analyzed by ABI 3730 Genetic Analyzer (Applied Biosystems). We randomly picked 5% of samples to undergo Sanger sequencing to ensure the genotyping success rate of the SNPs tested maintained more than 98% accuracy.

### Statistical analysis

To compare demographic characteristics (gender and age proportions) of the case and control groups, we performed the χ^2^ test and t-test using SPSS software (version 17.0). This study focused on two types of myopia, HM and EM. Therefore, we use a standard observed-expected χ^2^ test to evaluate the Harder-Weinberg equilibrium (HWE) of individual SNPs in the three groups (HM, EM, Control). After determining and accounting for the three different genotypes in both the patients and controls, *P* values were calculated using the Pearson’s χ^2^ test. Model-based (homozygous, heterozygous, dominant, and recessive) associations of the SNPs with HM and EM were analyzed by the χ^2^ test. Results of all statistical analyses were considered statistically significant at a *P* value < 0.05.

Haplotype blocks were defined by the Haploview software 4.2. Four SNPs in the *FGF10* region were located within a single haplotype block. LD values are expressed as D′ and r^2^, and odds ratio (OR) and 95% confidence interval (CI) were calculated for each haplotype using the SPSS 17.0. HaploReg v4.1 and RegulomeDB databases, two popular SNP functional annotation tools, were used to analyze the potential function of these SNPs.

## Results

### SNP analysis

In this study, we recruited 1768 unrelated subjects of whom 869 were HM patients (including 485 EM) and 899 were healthy controls. The average spherical refractive error in patients with HM was − 13.04 ± 6.43 DS (range, − 3.0 to − 31.0 DS) in the right eye (OD) and − 12.85 ± 6.39 DS (range, − 3.0 to − 30.0 DS) in the left eye (OS). The AL value in patients with HM was 29.68 ± 3.46 mm (range, 24.77 to 39.32 mm) and 29.51 ± 2.42 mm (range, 19.71 to 37.96 mm). The age of the patients ranged from 3 to 84 years old (41.60 ± 20.57 years), and male patients comprised 55.35% of the patient population. The age of the control subjects ranged from 15 to 85 years old (54.92 ± 19.13 years), and male subjects comprised 53.84% of the controls. Other demographic data are given in Table 1.

Four target SNPs were successfully genotyped, and the genotype distributions were within HWE in both case and control groups (*P* > 0.01). However, none of the four SNPs showed a positive association with HM (allelic *P* > 0.05, Table 2). Next, we performed an exploratory analysis to compare 485 patients with EM and controls. Results also showed no significant association between four SNPs and EM (*P* > 0.05, Table 3). Additionally, four genetic models were used to investigated other potential association between these SNPs and high myopia. In rs12517396, the frequency of AA was much lower in both HM and EM groups than that in control (0.6 and 0.2% vs. 1.6%, respectively). The recessive model suggested that rs12517396AA carriers had a decreased risk of HM and EM compared with rs12517396AC + CC carriers (*P* = 0.045, OR = 0.366, 95% CI = 0.131–1.020; *P* = 0.021, OR = 0.131, 95% CI = 0.017–0.996; respectively, Table 4). Very similar results have been found in the SNP of rs10462070. The recessive model suggested that rs10462070GG carriers had a decreased risk of HM and EM compared with rs10462070GA + AA carriers (*P* = 0.030, OR = 0.341, 95% CI = 0.123–0.942; *P* = 0.015, OR = 0.122, 95% CI = 0.016–0.159, respectively, Table 5). Additionally, rs12517396AA and rs10462070GG carriers showed same decreased risk of HM/EM compared with rs12517396CC and rs10462070AA carriers (*P* = 0.048, OR = 0.370, 95% CI = 0.133–1.033; *P* = 0.023, OR = 0.133, 95% CI = 0.018–1.020; *P* = 0.032, OR = 0.346, 95% CI = 0.123–0.956; *P* = 0.017, OR = 0.126, 95% CI = 0.017–0.954). However, after adjusting for multiple testing, rs12517396AA and rs10462070GG carriers only showed a marginally decreased tendency of HM/EM compared with rs12517396AC + CC and rs10462070GA + AA carriers or compared with rs12517396CC and rs10462070AA carriers (Tables 4 and 5). Furthermore, these genetic models were also applied to evaluate the association between two other SNPs and HM/EM. However, no significant association was found (data not shown).

**Table 2:** Association analysis between high myopia and 4 SNPs in a Han Chinese population

SNP	Chr.	Position	Gene	Major/minor allele	MAF	P-HWE		Allelic P^*^	OR (95% CI)	Corrected P^**^
					Case	Control	Case/Control			
rs1384449	5	44376958	*FGF10*	A/G	0.279	0.288	0.465/0.818	0.242	0.903 (0.761–1.071)	1
rs339501	5	44365531	*FGF10*	T/C	0.112	0.117	0.099/0.605	0.334	0.990 (0.780–1.257)	1
rs12517396	5	44359424	*FGF10*	C/A	0.110	0.115	0.053/0.470	0.328	0.989 (0.777–1.258)	1
rs10462070	5	44305647	*FGF10*	A/G	0.111	0.116	0.051/0.333	0.339	1.009 (0.794–1.283)	1

*SNP* = single nucleotide polymorphism, *Chr*. = chromosome, *MAF* = minor allele frequency, *HWE* = Hardy-Weinberg equilibrium, *OR* = odds ratio, *CI* = confidence interval

^*^Allelic *P* value has been adjusted for age and sex

^**^ Corrected *P* = Allelic *P* × 4 (the number of genotyped SNPs)

**Table 3:** Association analysis between extreme myopia and 4 SNPs in a Han Chinese population

SNP	MAF		P-HWE		Allelic P^*^	OR (95% CI)	Corrected P^**^
	EM	control	EM	Control			
rs1384449	0.270	0.288	0.591	0.818	0.309	0.909 (0.765–1.093)	1
rs339501	0.114	0.117	0.051	0.605	0.322	0.987 (0.763–1.277)	1
rs12517396	0.111	0.115	0.021	0.469	0.271	0.979 (0.755–1.270)	1
rs10462070	0.114	0.116	0.017	0.333	0.306	1.016 (0.785–1.315)	1

*SNP* = single nucleotide polymorphisms, *MAF* = minor allele frequency, *HWE* = Hardy-Weinberg equilibrium, *OR* = odds ratio, *CI* = confidence interval, *EM* = extreme myopia

^*^Allelic *P* value has been adjusted for age and sex

^**^ Corrected *P* = Allelic *P* × 4 (the number of genotyped SNPs)

**Table 4:** Association analysis between rs12517396 and HM/EM in 4 genetic models

Group	Genotype (n)			Genetic Model	OR (95% CI)	*P* ^*^	Corrected P^**^
	AA	AC	CC				
Control	14 (0.016)	178 (0.198)	707 (0.786)				
HM	5 (0.006)	182 (0209)	682 (0.785)	Homozygote	0.370 (0.133–1.033)	0.048	0.192
				Heterozygote	1.060 (0.841–1.337)	0.623	1
				Dominant	1.010 (0.804–1.267)	0.934	1
				Recessive	0.366 (0.131–1.020)	0.045	0.180
EM	1 (0.002)	106 (0.219)	378 (0.779)	Homozygote	0.133 (0.018–1.020)	0.023	0.092
				Heterozygote	1.114 (0.849–1.460)	0.435	1
				Dominant	1.042 (0.798–1.367)	0.761	1
				Recessive	0.131 (0.017–0.996)	0.021	0.084

Genotype analyses were conducted for the homozygote model (AA compared with CC), heterozygote model (AC compared with CC), dominant model (AA+AC compared with CC), and the recessive model (AA compared with AC + CC)

*HM* = high myopia, *EM* = extreme myopia, *OR* = odds ratio, *CI* = confidence interval

^*^ *P* value has been adjusted for age and sex

^**^ Corrected *P* = *P* × 4 (the number of genetic models)

**Table 5:** Association analysis between rs10462070 and HM/EM in 4 genetic models

Group	Genotype (n)			Genetic Model	OR (95% CI)	*P* ^*^	Corrected P^**^
	GG	GA	AA				
Control	15 (0.017)	178 (0.198)	706 (0.785)				
HM	5 (0.006)	183 (0.211)	681 (0.784)	Homozygote	0.346 (0.123–0.956)	0.032	0.128
				Heterozygote	1.067 (0.845–1.344)	0.590	1
				Dominant	1.001 (0.805–1.267)	0.932	1
				Recessive	0.341 (0.123–0.942)	0.030	0.128
EM	1 (0.002)	109 (0.225)	375 (0.773)	Homozygote	0.126 (0.017–0.954)	0.017	0.068
				Heterozygote	1.153 (0.881–1.509)	0.300	1
				Dominant	1.073 (0.823–1.400)	0.603	1
				Recessive	0.122 (0.016–0.925)	0.015	0.060

Genotype analyses were conducted for the homozygote model (GG compared with AA), heterozygote model (GA compared with AA), dominant model (GG + GA compared with AA), and the recessive model (GG compared with GA + AA)

*HM* = high myopia, *EM* = extreme myopia, *OR* = odds ratio, *CI* = confidence interval

^*^ *P* value has been adjusted for age and sex

^**^ Corrected *P* = *P* × 4 (the number of genetic models)

**Fig. 1:**
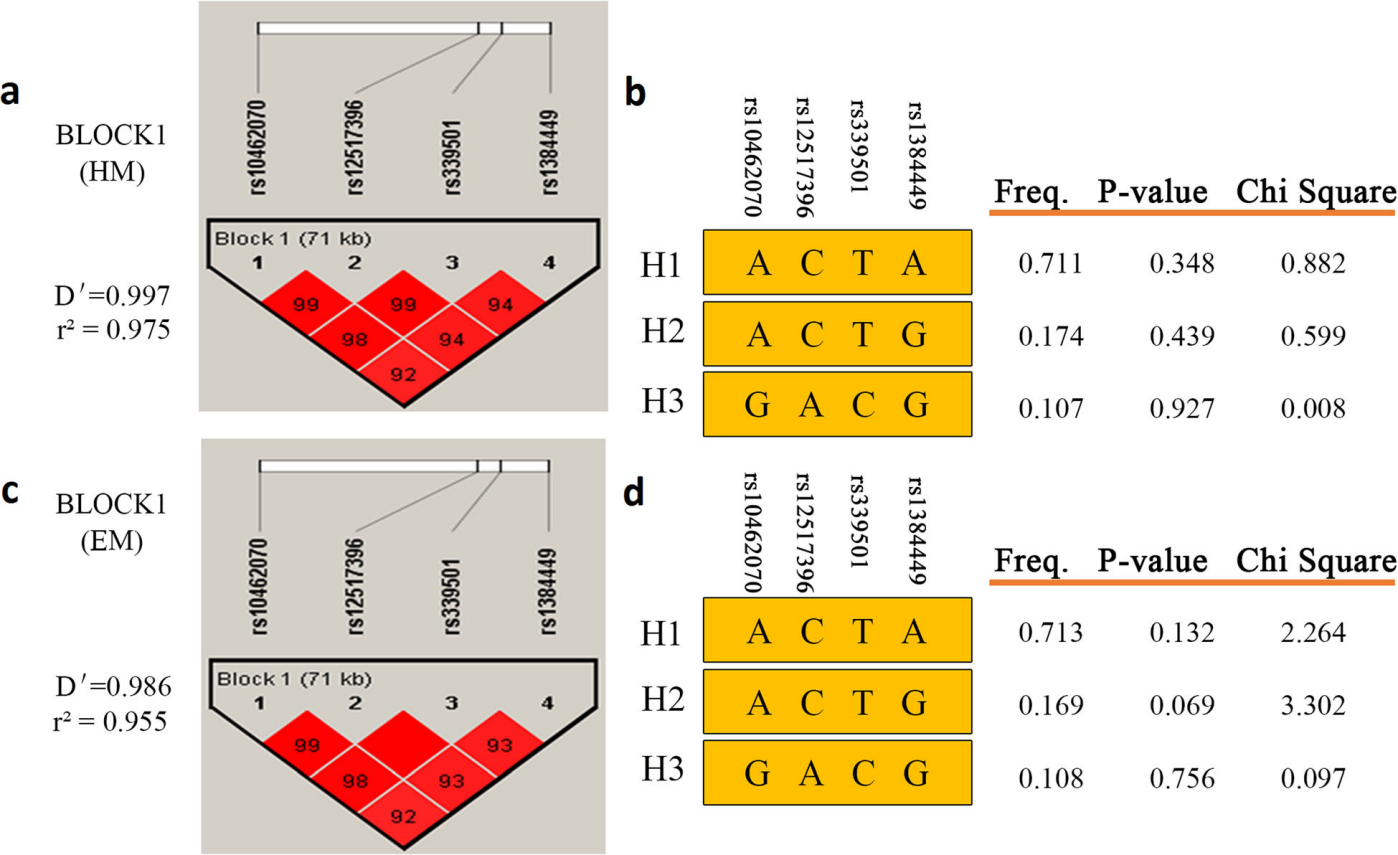
Linkage disequilibrium (LD) structure across rs10462070, rs12517396, rs339501 and rs1384449 region and results of haplotype-based association study (D’ values shown). **a** LD was measured using combined high myopia (HM) case and normal control data. The physical position of each singles nucleotide polymorphisms (SNP) is shown in the upper diagram. Each box provides estimated statistics of the coefficient of determination (D’), with darker shades representing stronger LD. **b** For HM, 3 haplotypes were observed, but no significant association was detected. **c** LD was measured using combined EM case and normal control data. **d** For extreme myopia (EM), 3 haplotypes were observed, but no significant association was detected

We then performed haplotype analysis using Haploview 4.2 software to examine the linkage disequilibrium (LD) structure of these SNPs in the *FGF10* gene. The four SNPs (rs1384449, rs339501, rs12517396 and rs10462070) were in the same LD block in both HM and EM groups (D′ =0.997, *r*^*2*^ = 0.975; D′ =0.986, *r*^*2*^ = 0.955; respectively, Fig. 1). However, all of the haplotypes showed no significant association between HM/EM and control groups (*P* > 0.05, Fig. 1).

In order to better understand the annotation information of SNPs in the public databases, we explored potential biological functions of 4 SNPs (rs12517396, rs10462070, rs10512851 and rs16901825) in the RegulomeDB and HaploReg v4.1 database (Table 6). Interestingly, in RegulomeDB, rs12517396, rs10462070 and two SNPs (rs16901825 and rs10512851) were in the same LD block in 100 healthy Chinese Genomes group (D′ =0.86, *r*^*2*^ = 0.95; D′ =0.86, *r*^*2*^ = 0.95; respectively). Rs16901825 showed a likely evidence of affecting binding with STAT3 and CEBPB protein (Score = 3a) and rs10512851 showed a minimal binding evidence with binding STAT3 protein.

**Table 6:** Annotation function information for rs12517396, rs16901825, and rs10512851

SNP	LD-r^2^	LD-D′	ORF	Score of Regulome BD	Function	Binding protein	Regulatory motifs altered
rs12517396	1	1	no	6	Histone modification	no	Bbx, Gfi1, HDAC2, HMG-IY, Hbp1, Nanog, Nkx6, SOX
rs10462070	1	1	no	no date	no	no	AIRE, Ets-disc1, Hnf6, PlZF, pou3f2
rs16901825	0.95	0.86	no	3a	Histone modification	STAT3	ATF3
rs10512851	0.95	0.86	no	5	Chromatin structure	STAT3, CEBPB	Foxp1

Scores of Regulome DB have different meanings. 3a means this SNP is likely to be located in a trans factor binding site and deoxyribonuclease peak area. Five means this SNP is less likely to be located in a trans factor binding site or deoxyribonuclease peak area. Six means others

*SNP* = single nucleotide polymorphisms, *ORF* = open reading frame

Rs12517396 could connect with many motifs (such as Bbx, Gfi1_1, Gfi1_3, Gfi1b, HDAC2_disc6, HMG-IY_2, Hbp1, Nanog_disc2, Nkx6-1_2, SOX_18 and Sox_2), and it was more likely to bind with HDAC2_disc6 motif (match on: DRRRRARRAARRRMW) and NKx6-1_2 (RVWWWWTAATKAMYBBB) motif (Ref =0, Alt =11.6; Ref =3.5, Alt =12.4; respectively). In addition, rs10462070 alters the regulatory motifs of transcription factors AIRE_1, Ets_disc1, HNF6, and Pou3f2_1, and it was more likely to bind with PLZF (Ref =6.7, Alt = − 4.3) motif which matches the following protein sequence: RMAYWRDYMMWRMTTTAVMDYMVRWMBAV.

## Discussion

Earlier studies supported that the family of FGFs may be risk factors for myopia. The SNP of rs339501 in the *FGF10* gene has been reported to be associated with EM but not with HM in a population in Taiwan, China [24]. Yoshida et al. found that 3 SNPs (rs339501, rs12517396 and rs10462070) in *FGF10* that also showed significant associations with EM in the Japanese population [23]. Furthermore, the FDM mouse model verified that the mRNA level of *FGF10* was significantly increased in FDM eyes [24].

In this study, we genotyped four SNPs, including rs1384449, rs339501, rs12517396 and rs10462070, in *FGF10* and tested their relationship with HM and EM in a western Chinese population. The frequency of the rs339501TT allele was higher in EM/HM groups than that in control group (79.2%/78.5% vs. 77.8%), which showed a similar trend with the Japanese population but opposite trend compared with the population in Taiwan, China. In addition, it is interesting to note that the minor allele homozygote of rs12517396 (AA) and rs10462070 (GG) showed a protective effect with respect to the susceptibility of HM and EM in our study. This is also very similar to the results of the study of Japanese population. The frequencies of these two genotypes were both much lower in the EM group (0.2%) than that in the HM group (0.6%), suggesting that the protective effect of these genotypes might be stronger against EM than HM. However, the sample size needs to be increased in order to confirm a more accurate and clearer relationship between these genetic variants and the disease.

Rs12517396 was located in the promoter and enhancer area of *FGF10* and it might regulate the binding between motif (HDAC2_disc6 and NKx6-1_2) and DNA promoter area. Even though rs10462070 was not found possible to be binding proteins, it can alter the regulatory motifs of transcription factors such as PLZF. All of these suggested that it may regulate gene expression in a cis or trans fashion. Additionally, rs12517396, rs10462070 and two other SNPs (rs16901825, rs10512851) were in the same LD block (*r*^*2*^ > 0.85) in the 100 Chinese Genomes group according to the RegulomeDB database. Rs16901825 and rs10512851 were also located in the promoter area of *FGF10* and both of them could bind with STAT3 (Signal transducers and activators of transcription). STAT3, localized to the eye from the embryonic stage, play a central role in mediating cell differentiation and survival signals [22]. In addition, the STAT3 pathway also induces the alternative activation of macrophages and vascular proliferation which could cause blinding eye disease including high myopia [25]. Previous study also showed that retinoic acid could affect the development of myopia by regulating TGF-β pathway and the expression of FGF10 [23]. Further evidence showed that STAT3 play a crucial role in the regulation of TGF-β pathway [26, 27]. Taken together, these suggest that rs12517396 was associated with HM/EM susceptibility probably through the STAT3 TGF-β pathway.

## Conclusions

In conclusion, we found that rs12517396 and rs10462070 in *FGF10* have marginal associations with HM and EM (especially with EM) under the recessive model in this western Chinese population. The susceptible effect of rs12517396 and rs10462070 to extreme myopia observed in our study, however, should be validated in other independent cohorts. Rs12517396 might participate in the STAT3 and TGF-β pathway to influence the development of myopia. Furthermore, to avoid filtering real myopia genes, the role of *FGF10* in the pathogenesis of myopia requires more refinement in both animal models and human genetic epidemiologic studies.

## Data Availability

All data generated or analyzed during this study are included in this published article.
